# The Origin of the ‘*Mycoplasma mycoides* Cluster’ Coincides with Domestication of Ruminants

**DOI:** 10.1371/journal.pone.0036150

**Published:** 2012-04-27

**Authors:** Anne Fischer, Beth Shapiro, Cecilia Muriuki, Martin Heller, Christiane Schnee, Erik Bongcam-Rudloff, Edy M. Vilei, Joachim Frey, Joerg Jores

**Affiliations:** 1 Molecular Biology and Biotechnology Department, International Centre for Insect Physiology and Ecology, Nairobi, Kenya; 2 Biotechnology Department, International Livestock Research Institute, Nairobi, Kenya; 3 Department of Biology, Pennsylvania State University, University Park, Pennsylvania, United States of America; 4 Institute of Molecular Pathogenesis, Friedrich-Loeffler-Institute, Jena, Germany; 5 Department of Animal Breeding and Genetics, Swedish University of Agricultural Sciences, Uppsala, Sweden; 6 Institute of Veterinary Bacteriology, University of Bern, Bern, Switzerland; Miami University, United States of America

## Abstract

The ‘*Mycoplasma mycoides* cluster’ comprises the ruminant pathogens *Mycoplasma mycoides* subsp. *mycoides* the causative agent of contagious bovine pleuropneumonia (CBPP), *Mycoplasma capricolum* subsp. *capripneumoniae* the agent of contagious caprine pleuropneumonia (CCPP), *Mycoplasma capricolum* subsp. *capricolum*, *Mycoplasma leachii* and *Mycoplasma mycoides* subsp. *capri*. CBPP and CCPP are major livestock diseases and impact the agricultural sector especially in developing countries through reduced food-supply and international trade restrictions. In addition, these diseases are a threat to disease-free countries. We used a multilocus sequence typing (MLST) approach to gain insights into the demographic history of and phylogenetic relationships among the members of the ‘*M. mycoides* cluster’. We collected partial sequences from seven housekeeping genes representing a total of 3,816 base pairs from 118 strains within this cluster, and five strains isolated from wild *Caprinae*. Strikingly, the origin of the ‘*M. mycoides* cluster’ dates to about 10,000 years ago, suggesting that the establishment and spread of the cluster coincided with livestock domestication. In addition, we show that hybridization and recombination may be important factors in the evolutionary history of the cluster.

## Introduction

Members of the genus *Mycoplasma* belong to the most important bacterial livestock pathogens worldwide. Of particular importance are *Mycoplasma mycoides* subsp. *mycoides* (*Mmm*) and *Mycoplasma capricolum* subsp. *capripneumoniae* (*Mccp*), two members of the ‘*Mycoplasma mycoides* cluster’ [1], which are responsible for contagious bovine pleuropneumonia (CBPP) and contagious caprine pleuropneumonia (CCPP), respectively. Both diseases cause significant losses in livestock, in particular in Africa and Asia, and are a threat to disease-free countries.

The causative agent of CBPP was cultivated and characterized for the first time by Nocard and Roux in 1898 [2]. CBPP was first recorded in Europe and was introduced into Africa, North America, Australia, and New Zealand during the colonial time period in the 18^th^ and 19^th^ centuries via livestock movement [3]. Today, CBPP is present in sub-Saharan Africa and suspected in Asia.

CCPP was first described in Algeria in 1873 [4]. Its highly contagious nature was acknowledged after an outbreak in South Africa in 1881, which was traced back to the importation of infected goats from Turkey. CCPP is a significant disease of goats in Africa, the Middle East and Western Asia and causes mortalities of up to 80%.

Besides the causative agents of CCPP and CBPP, the ‘*M. mycoides* cluster’ encompasses additional pathogens, including the bovine pathogen *M. leachii* [5] and the small ruminant pathogens *M. mycoides* subsp. *capri (Mmc)* and *M. capricolum* subsp. *capricolum (Mcc)*. Diseases caused by members of the cluster are characterized by clinical symptoms including pneumonia, mastitis, septicaemia, meningitis, wound infections, and arthritis.

Several studies have attempted to resolve the evolutionary relationships between the members of the ‘*M. mycoides* cluster’ [6], [7], [8], or to infer the evolutionary history of single members within the cluster [9], [10], [11]. However, despite the recent publication of complete genome data from single isolates belonging to the different lineages [12], a comprehensive overview of the evolutionary history of the ‘*M. mycoides* cluster’ and genetic relationship between populations is still lacking. Here, we partially sequence seven housekeeping genes from all members of the ‘*M. mycoides* cluster’, spanning their geographic distribution and isolated over the last 100 years. We use these data to infer the recent demographic and evolutionary history of these lineages, to estimate the timing of the origin of each member of the cluster, and correlate the estimated demographic history of the pathogens with that of their hosts.

## Results

### Genetic relationship between populations

**Figure 1:**
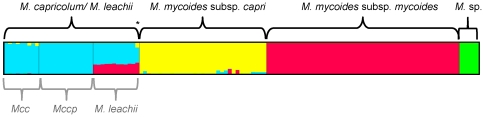
Population structure and phylogenetic relationship of the ‘*M. mycoides* cluster’ and five non-species assigned *Mycoplasma* strains. The 4 populations revealed by the STRUCTURE analysis using the linkage model and 7 housekeeping sequences are displayed on the top of the figure and marked with different colours. The ancestral parts of each strain are displayed in vertical lines. Subpopulations of the *M. capricolum*/*M. leachii* population are displayed below.

According to our STRUCTURE analysis, the *Mycoplasma* strains investigated here fall into four distinct populations, three of which belong to the ‘*M. mycoides* cluster’. These three are *Mmm*, *Mmc*, and *M. capricolum/M. leachii*. The fourth population consist of five strains of an unassigned *Mycoplasma* species (*M.* sp.) that were isolated from wild *Caprinae*. *M. leachii* appears to be a hybrid between *Mmm* and *M. capricolum*, with all 11 individuals showing at least 30% ancestry from *Mmm* and the remaining from *M. capricolum* (Figure 1). Strain B144P, isolated from cattle and formerly assigned as *Mycoplasma* sp. serogroup L [13], shows additional evidence for hybridization. This strain clusters with *M. leachii* and shows 60% ancestry from *M. capricolum*, 30% from *Mmm*, and 10% from *Mmc*.

In a first phylogenetic analysis including all 123 *Mycoplasma* strains, the five strains of unassigned *M.* sp. clustered together as an outgroup to the ‘*M. mycoides* cluster’. Nevertheless the use of distant outgroups can lead to a distortion of the phylogeny. We plotted transition and transversion rates versus genetic distances (Figure S1). As expected, the observed number of transitions is higher than that of transversions among strains of the ‘*M. mycoides* cluster’ (ingroup). However, the observed number of transversions is higher than that of transitions for pairwise comparisons between the outgroup and the ingroup species, which implies that substitution saturation has occurred during the divergence between the outgroup and the ingroup. We therefore excluded *M.* sp. from the phylogenetic analysis. Instead we assumed equal evolutionary rates across all branches and used midpoint rooting for drawing the estimated trees.

The midpoint rooted maximum likelihood phylogeny estimated using the concatenated data set agrees with previously reported data [8] in that the ‘*M. mycoides* cluster’ is divided into two subclusters, one comprising *Mmc* and *Mmm*, the second *M. capricolum* and *M. leachii* (Figure S2). In addition, this analysis shows strong statistical support for the monophyly of the three ‘*M. mycoides* cluster’ populations defined above. Here however, *M. leachii* clusters separately from *M. capricolum* with strong statistical support. The subspecies of *M. capricolum*, *Mccp* is monophyletic, while *Mcc* is found to be paraphyletic. Phylogenies estimated for each locus independently varied in topology (Figure S3), and many were incongruent with the phylogenetic tree estimated from the concatenated data set. The incongruences in phylogenetic trees may be explained by hybridization between populations of the cluster, as identified in the structure analysis.

### Population demography

We computed three different summary statistics (θ_w_, Tajima's D and Fu's Fs) to characterize the patterns of genetic diversity within the four populations from the structure analysis and treating the hybrid *M. leachii* as a fifth, separate subpopulation (Table 1). In addition, we estimated recombination rates per bp in three (sub)populations, *Mmc*, *M. capricolum* and *M. leachi* (Table 2). We did not estimate recombination in either *Mmm* or in the population of unassigned *Mycoplasma* strains because of their very low genetic diversity.

**Table 1:** Summary statistics for seven genes in five (sub)populations.

	θ_w_ (%)					Tajima's D					Fu's Fs				
	*Mmc*	*M. c.*	*M. leachii*	*Mmm*	*M.* sp.	*Mmc*	*M. c.*	*M. leachii*	*Mmm*	*M.* sp.	*Mmc*	*M. c.*	*M. leachii*	*Mmm*	*M.* sp.
*adk*	1.52	0.57	1.85	n.a.	n.a.	−1.13	−1.03	−2.17*	n.a.	n.a.	−5.29*	−0.79	3.63	n.a.	n.a.
*gmk*	1.19	0.82	0.93	0.05	0.48	−1.19	0.01	−1.99*	−1.1	−1.12	−5.41*	0.48	1.45	−1.64	2.64
*gyrB*	2.23	0.70	0.34	0.04	0.41	−0.28	−1.17	−1.43	−1.1	−1.12	−2.09	−2.62	−1.36	−1.64	0.64
*pdhC*	1.15	0.66	0.33	0.05	n.a	−0.61	−1.51	−1.83*	−1.1	n.a.	−4.17	−3.24	0.33	−1.64	n.a.
*pgi*	1.13	0.81	0.40	0.05	0.19	−2.01*	−0.68	−1.63	−1.1	−0.97	−9.46*	−1.88	0.95	−1.64	1.04
*recA*	1.30	0.30	0.12	n.a.	n.a.	−1.10	−0.59	−1.45	n.a.	n.a.	−7.25*	−2.28	−1.33	n.a.	n.a.
*rpoB*	1.58	0.93	0.10	0.03	0.14	−1.23	−0.58	−1.45	−1.1	−0.97	−5.55*	0.56	−1.33	−1.64	1.04

θ_w_ is a measure of genetic diversity, Tajima's D and Fu's Fs are two summaries of allele frequencies.

*Mmc* - *Mycoplasma mycoides* subsp. *capri*, *M. c.* - *Mycoplasma capricolum* (both subsp.), *Mmm* - *Mycoplasma mycoides* subsp. *mycoides*, *M.* sp. - unassigned *Mycoplasma* species,

* Significant values p<0.05.

**Table 2:** Population recombination rate estimates (ρ) for three (sub)populations.

	θ_w_ (%)			ρ = 2*N_e_*r		
	*Mmc*	*M. capricolum*	*M. leachii*	*Mmc*	*M. capricolum*	*M. leachii*
*adk*	1.52	0.57	1.85	0.006	0	0
*gmk*	1.19	0.82	0.93	0.068	0	0
*gyrB*	2.23	0.70	0.34	0.048*	0.056	0
*pdhC*	1.15	0.66	0.33	0.045	0.008	0
*pgi*	1.13	0.81	0.4	0.004	0.018	0
*recA*	1.3	0.30	0.12	0.066	0.009	0.031
*rpoB*	1.58	0.93	0.1	0.117*	0.006	0.100

*Mmc*-*Mycoplasma mycoides* capri,

* Significant values p<0.05.

*Mmc* shows the highest genetic diversity (θ_w_ = 0.014). In addition, the test for recombination shows that significant levels of recombination occur in two genes within *Mmc* (Table 2). Levels of genetic diversity in the four other populations are low and we find no evidence for recombination. However, the sample sizes for several of the populations are very small, and it is possible that additional data may influence these results. In particular, as our analyses have revealed *M. leachii* as a likely hybrid, we should not exclude the possibility that recombination may be more important than this simple analysis suggests [14].

For each locus, we assessed how well a null model of constant population size and random mating fits each population by estimating Tajima's D and Fu's Fs statistics. Negative values reflect an excess of rare alleles, which can be either due to a demographic scenario such as population expansion or to positive selection. Three loci have a significantly negative Tajima's D for *M. leachii*, with negative values (although not significant) for the other four genes. *Mmc* also deviates from the null model in that Fu's Fs values are significantly negative for five of the seven loci, with negative Tajima's D values for all loci, although significantly negative for only one locus. Since it is unlikely that all the housekeeping genes used in this study are under positive selective pressure, we favour population expansion as an explanation of this departure from the null model.

**Figure 2:**
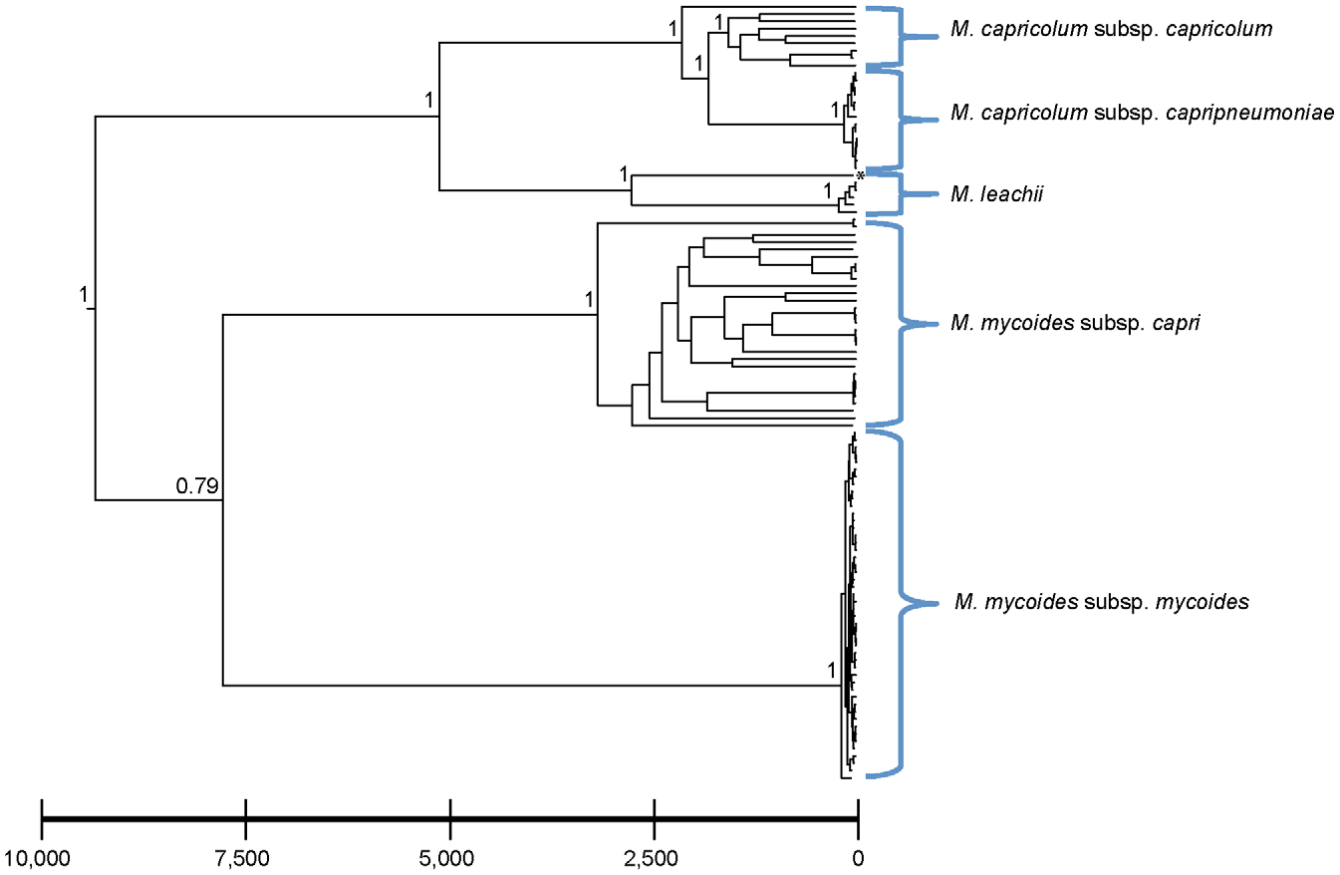
Maximum credibility tree resulting from the BEAST analysis of the concatenated sequence alignment, as described in the main text. Bayesian posterior probabilities are provided at the major nodes in the tree as a measure of support for clustering of the distinct strains. The scale is given in years before present.

The maximum clade credibility tree resulting from the combined BEAST analysis of the 110 strains for which a year of isolation was available (excluding the five unassigned *M.* sp. strains) is shown in Figure 2. All strains fall into monophyletic clusters with strong statistical support. The 110 strains included in this analysis shared a common ancestor ca. 10,000 years ago, while *Mmc*, *M. capricolum* and *M. leachii* all share common ancestors between 2,300 and 4,500 years ago (Table S3). *Mccp*, the agent of CCPP, shared a common ancestor between only 56 and 490 years ago.

## Discussion

The present study is based on 118 strains belonging to the ‘*M. mycoides* cluster’ and represents the largest comparative study of the ‘*M. mycoides* cluster’ to date. Strikingly, the estimated origin of the ‘*M. mycoides* cluster’ appears to coincide with the onset of domestication of small and large ruminants about 10,000 years ago [15], [16], [17], [18]. Domestication was associated with both the establishment of large ruminant populations and the herding of mixed species. Both of these factors may have contributed to creating environmental conditions favouring the spread and diversification of the ‘*M. mycoides* cluster’ as the organisms adapted to different hosts.

It has been shown previously that genetic exchange between different *Mycoplasma* species sharing the same host is possible within the genus *Mycoplasma* [19], [20]. However, our study provides evidence for genetic exchange between and within *Mycoplasma* populations having different primary hosts. For example, *M. leachii* appears to be a hybrid of *Mmm* and *M. capricolum*. In addition, the bovine strain B144P, which had been identified as *Mycoplasma* serogroup L [13], actually belongs to the *M. leachii* hybrid subpopulation, but contains ancestry from all ‘*M. mycoides* cluster’ populations defined in this study (*Mmc*, *Mmm* and *M. capricolum*). Since species belonging to the ‘*M. mycoides* cluster’ are obligate parasites and require the host for survival, any horizontal gene transfer or recombination must have happened within the host. Recent reports have shown that species of the ‘*M. mycoides* cluster’ can survive in non-primary hosts. For example, the bovine pathogens *Mmm* and *M. leachii* (Table S1) have been isolated from goats [21], and caprine pathogens *M. capricolum* or *M. mycoides* subsp. *capri* have been isolated from cattle (Table S1) [22]. Other studies reported the isolation of different ‘*M. mycoides* cluster’ members from single, diseased individuals [23], [24], supporting the idea that the host itself might act as a hybridization oven for new ‘*M. mycoides* cluster’ variants. Mixed herding and pastoralist practices have been common in the past and remain widespread in Africa. These provide the opportunity for hosts to become infected with pathogens from other hosts. The resulting co-infection may facilitate the exchange of genetic material between pathogens.

The five strains of unassigned *Mycoplasma* sp. that were isolated independently from various *Carprinae* hosts on various continents over several years represent a distinct population (Table S1), related but not belonging to the ‘*M. mycoides* cluster’. We hypothesize that these isolates represent a novel species of *Mycoplasma* closely related to the ‘*M. mycoides* cluster’ that evolved in wild *Caprinae* [25]. However, thorough analyses of both biochemical and genomic traits are required to confirm this hypothesis. Of particular interest would be the comparison of diseases patterns caused by the different strains in their respective hosts. Our MLST typing scheme is a useful tool to characterize not only *Mycoplasma* isolates belonging to the ‘*M. mycoides* cluster’ but also to type closely related mycoplasmas.

## Materials and Methods

### Strains and samples

We collected data from 123 *Mycoplasma* strains, including 50 *Mmm*, 33 *Mmc*, 11 *M. leachii*, nine *Mcc*, 14 *Mccp*, five unassigned *Mycoplasma* strains isolated from wild *Caprinae* in different continents, and one strain of *Mycoplasma* previously typed as serogroup L strain. Sequences from three fully annotated mycoplasma genomes from GenBank (GM12, Accession number CP002027; ATCC27343, Accession number NC_007633; and PG1, Accession number NC_005364) were part of our data collection. The full data set comprised strains from Europe, America, Australia, Asia, and Africa that had been collected during the period spanning 1931–2005. Additional information about the strains comprising the full data set, including country and year of isolation, are provided in Table S1.

### Target amplification and sequencing

We selected 7 housekeeping genes, which were used previously for MLST analysis in other bacteria [7], [26], [27], [28]: adenylate kinase (*adk*), guanylate kinase (*gmk*), DNA gyrase subunit B (*gyrB*), dihydrolipoamide S-acetyltransferase (*pdhC*), glucose-6-phosphate isomerase (*pgi*), recombination protein (*recA*), and DNA-directed RNA polymerase beta chain (*rpoB*). About 500 bp of each housekeeping gene were sequenced representing a total of 3816 bp per strain. The genes used are randomly distributed in the 1 MB genome of the *Mmm* type strain PG1 (Figure S4). PCR was carried out in duplicate (50 µl reaction volume) using GoTaq® Green master mix (Promega, USA) polymerase according to manufacturers' instructions, and primers listed in Table 2. Five to 20 ng of genomic DNA were used as template per reaction, annealing temperatures are provided in Table S2. PCR products were purified using QIAquick PCR purification kit (QIAGEN, Germany) and sequenced by Macrogen Inc. (Seoul, Korea), Agowa GmbH (Berlin, Germany), or StarSeq GmbH (Mainz, Germany). Sequence traces were assembled using the Staden program package (http://staden.sourceforge.net/) and trimmed (Table S2) by eye. Sequences are available in GenBank (Accession numbers JQ673623–JQ674483).

### Demographic analysis

To infer recent demographic trends among members of the cluster, we calculated common summary statistics for the entire data set and for each member of the cluster separately using DNAsp v5 [29]. We estimated nucleotide diversity (θ_w_) [30] and summaries of allele frequencies (Tajima's D, Fu's Fs) [31], [32], with significance obtained by comparing the observed values to 1000 simulated data sets.

### Recombination rates

In order to quantify the extent of homologous recombination within populations, we estimated population recombination rates using an extension of the composite–likelihood approach described before [33] and implemented in the program LDhat [34]. This method uses a finite-site model of substitution, which is more appropriate for bacterial evolution than the infinite sites model. Indeed, in bacteria, the rate of substitution is sufficiently high that some sites may have experienced multiple mutations in the history of the sample. We estimated the recombination rate ρ = 2*N_e_*r (where r is the per base rate of initiation of recombination and *N_e_* the effective population size). We tested for significance of recombination using the permutation test available in LDhat.

### Population structure

We estimated population structure using the linkage model in STRUCTURE v2.3.2 [35], [36]. To perform this test, we converted MLST sequence data from Extended FASTA Format into the Structure Format using *xmfa2struct* (available from http://www.xavierdidelot.xtreemhost.com/clonalframe.htm). This Bayesian approach uses multilocus genotypic data to define a set of populations with distinct allele frequencies, and assigns individuals/strains probabilistically to defined populations without prior knowledge of sampling location or sampled host. This program identifies admixed individuals/strains and gives an estimate of percent ancestry from ancestral population for each individual/strain. We performed three replications of the test, in which we initially discarded 10,000 Markov Chain Monte Carlo (MCMC) iterations as *burn-in* and kept the subsequent samples from 20,000 MCMC iterations for analysis. We tested values of *K* between 1 and 7, where *K* is the number of inferred populations. The results of the three independent runs were averaged for each *K* value to determine the most likely model, *i.e.* the one with the highest likelihood. Results were plotted using Distruct [37].

### Phylogenetic analysis and estimates of divergence times

We first tested whether *M.* sp. was a suitable outgroup for our phylogenetic analyses by estimating substitution saturation. We used the program DAMBE [38] to plot pairwise transition and transversion distances versus total genetic distance. We inferred the phylogenetic history of each housekeeping gene separately using the Bayesian approach implemented in MrBayes v3 [39]. We used the program jmodeltest1.0 [40] to select the best fitting model of nucleotide substitution, which, for each data set, was the Generalized Time Reversible model with invariant sites and gamma-distributed rate heterogeneity (GTR+I+G) [41].

For each gene and the concatenated data set, we used MrBayes to estimate four independent MCMC chains (one cold and three hot), each running for ten million iterations with samples drawn every 1000 iterations. We removed the first 10% of each run to allow for burn-in, and assessed convergence using the program Tracer v1.4.1 (http://tree.bio.ed.ac.uk/software/tracer/). The majority consensus trees were drawn using Figtree v1.3.1 (http://tree.bio.ed.ac.uk/software/figtree/).

We performed two additional analyses of the phylogenetic history of the concatenated data set. First, we estimated a phylogeny for all the sequences concatenated and all the strains. Initially, we used MrBayes and assumed the GTR+G+I model of nucleotide substitution, with separate evolutionary models assigned to each locus. We had difficulties reaching convergence of the Markov Chains in our analyses using a Bayesian framework. We therefore estimated a maximum likelihood phylogeny for all populations using PhyML 3.0 [42]. To assess statistical support for the resulting phylogeny, we performed 1000 bootstrap replicates assuming GTR+G+I model of nucleotide substitution. Secondly, we performed a molecular clock analysis of 110 strains, representing all populations and all loci, but excluding strains belonging to the population of strains of an unassigned *Mycoplasma* species, as well as the eight strains for which no year of isolation was available. We used the flexible Bayesian phylogenetic analysis package BEAST v1.6.1 [43], which allowed us to estimate the time of the divergence between the populations within the ‘*M. mycoides* cluster’. Here again, the GTR+G+I model proved to be an over parameterization of the data, and convergence of these particular parameters could not be achieved. For subsequent analyses, we used the HKY+G+I model, with different transition/transversion ratios, gamma parameters, and proportion of invariant sites estimated for each of the seven loci, but all loci informing the same tree. We assumed a strict molecular clock, with the evolutionary rate estimated using the collection date of each of the isolated sequences, and an internal, normally distributed calibration in which the age of the *Mmm* lineage is estimated to lie within a 95% confidence interval spanning 150–250 years ago [3], [8], [44]. Independent evolutionary rates were estimated for each of the seven loci. To account for potential structure among the different populations, we used the flexible Bayesian Skyline coalescent model [45]. Four BEAST analyses were run for 100 million iterations each, with trees and parameter values drawn from the posterior sample every 10,000 iterations. Chains were evaluated for convergence using Tracer. The first 10% of samples from each run were discarded as burn-in, and the remainder combined. The maximum clade credibility tree was estimated from the combined posterior sample of trees using TreeAnnotator v1.6.1 (http://beast.bio.ed.ac.uk/TreeAnnotator).

## Supporting Information

Figure S1 **Plot of transitions (blue crosses) and transversions (green triangles) versus genetic distance (Generalized Time Reversible model (GTR)) for seven concatenated sequences.**
(TIF)

Figure S2 **Mid-point rooted phylogenetic tree displaying the phylogentic relationship of the ‘** ***M. mycoides*** **cluster’.** The colour code used in Figure 1 was used to display the strain designation to different populations. The bootstrap values are displayed.
(TIF)

Figure S3 **50% majority consensus tree for each of the seven partial gene sequences as estimated with MrBayes under the GTR+G+I substitution model.**
(TIF)

Figure S4 **Genomic location of MLST target genes based on the PG1 genome.**
(TIF)

Table S1 Strains used in this study.
(DOC)

Table S2 Target genes, primer sequences and trimming region used for MLST of the *‘M. mycoides cluster’*.
(DOC)

Table S3 Bayesian MCMC estimates of time to the most recent common ancestor in years before present.
(DOC)
