# Supplementary figures and images for: The Origin of the ‘Mycoplasma mycoides Cluster’ Coincides with Domestication of Ruminants
Source: PLoS One. 2012 Apr 27;7(4):e36150. doi: 10.1371/journal.pone.0036150 (PMC3338596; doi:10.1371/journal.pone.0036150)

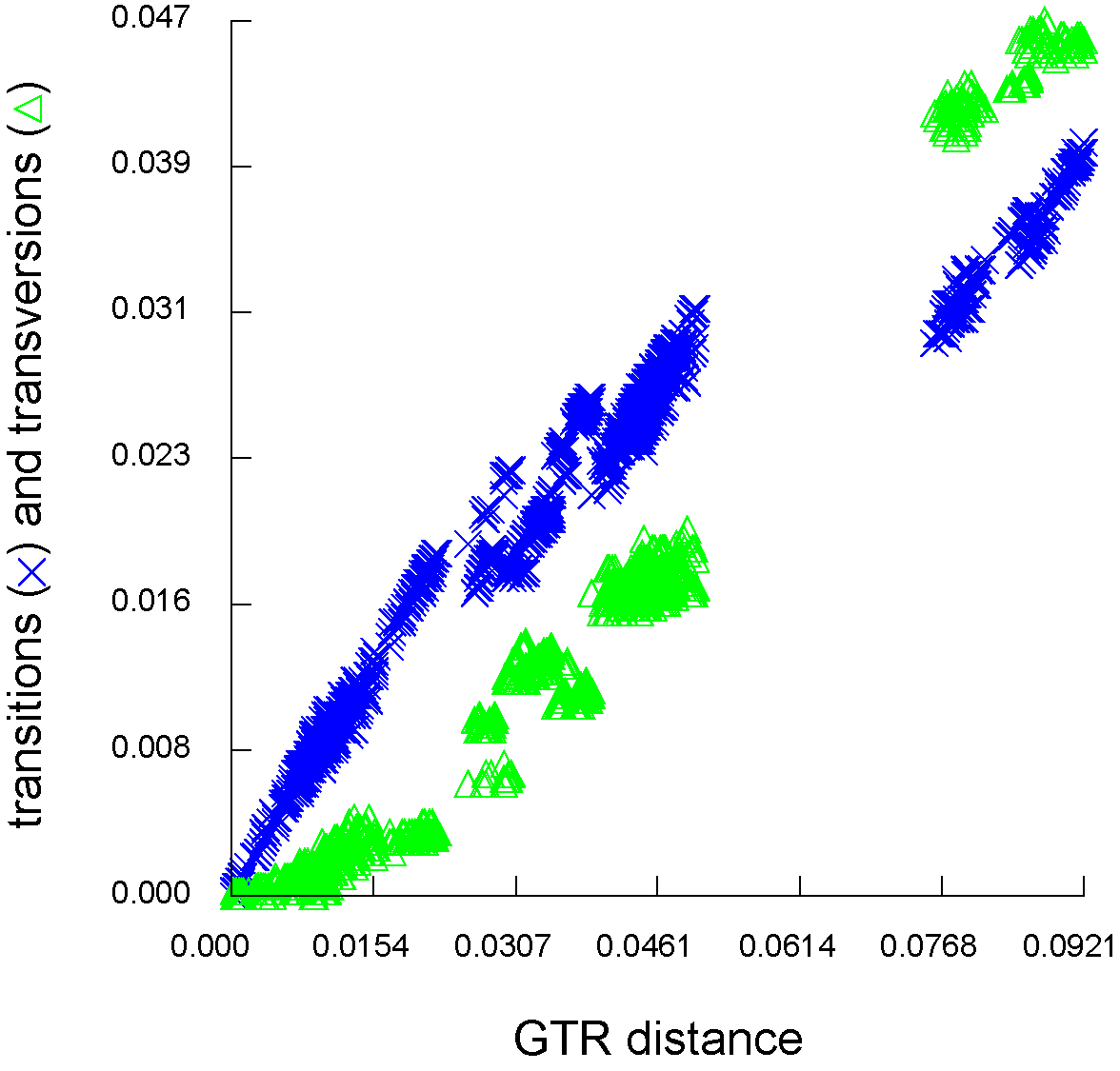

Supplement: Figure S1 — Plot of transitions (blue crosses) and transversions (green triangles) versus genetic distance (Generalized Time Reversible model (GTR)) for seven concatenated sequences. (TIF) [file pone.0036150.s001.tif]

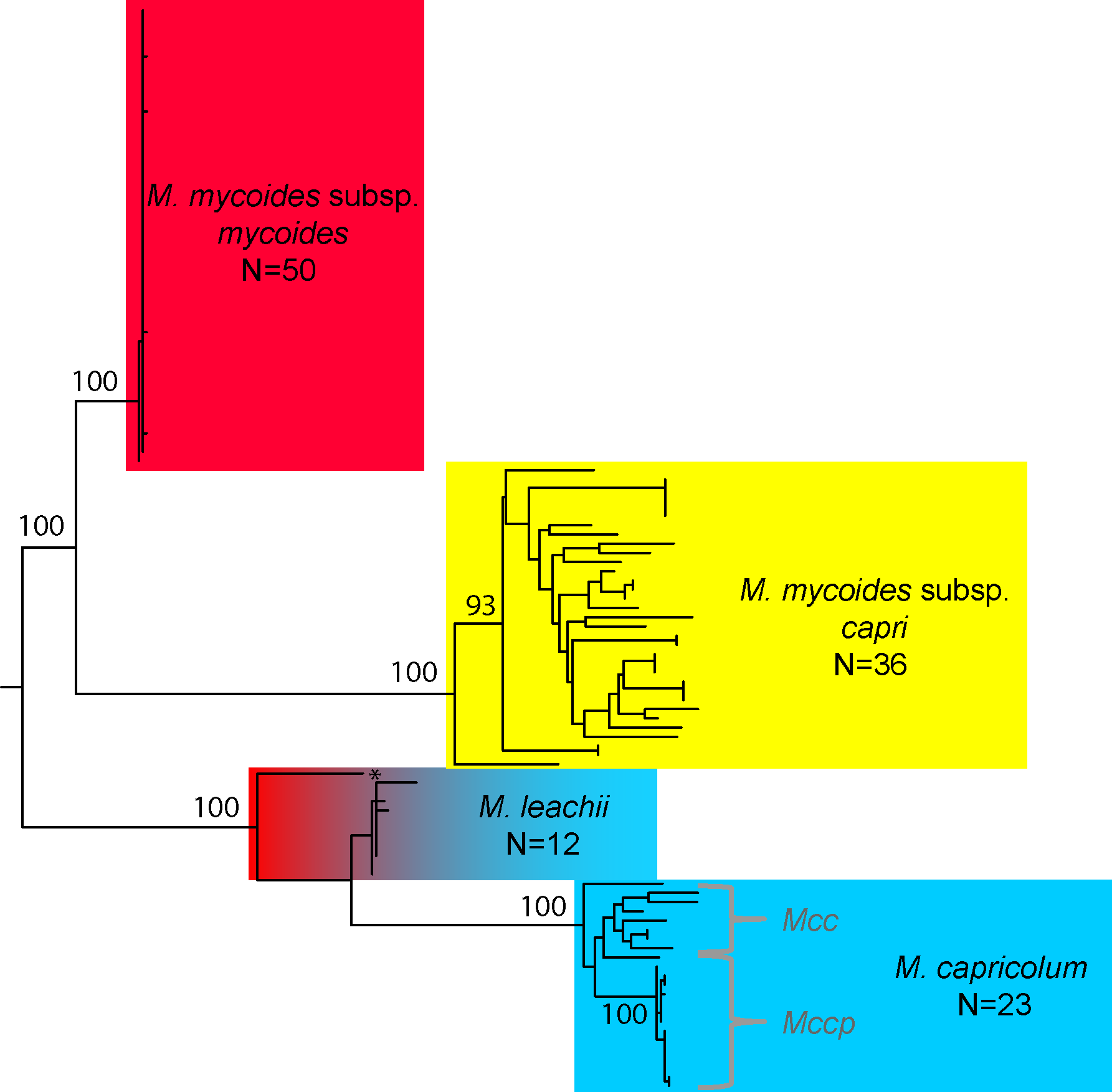

Supplement: Figure S2 — Mid-point rooted phylogenetic tree displaying the phylogentic relationship of the ‘ M. mycoides cluster’. The colour code used in Figure 1 was used to display the strain designation to different populations. The bootstrap values are displayed. (TIF) [file pone.0036150.s002.tif]

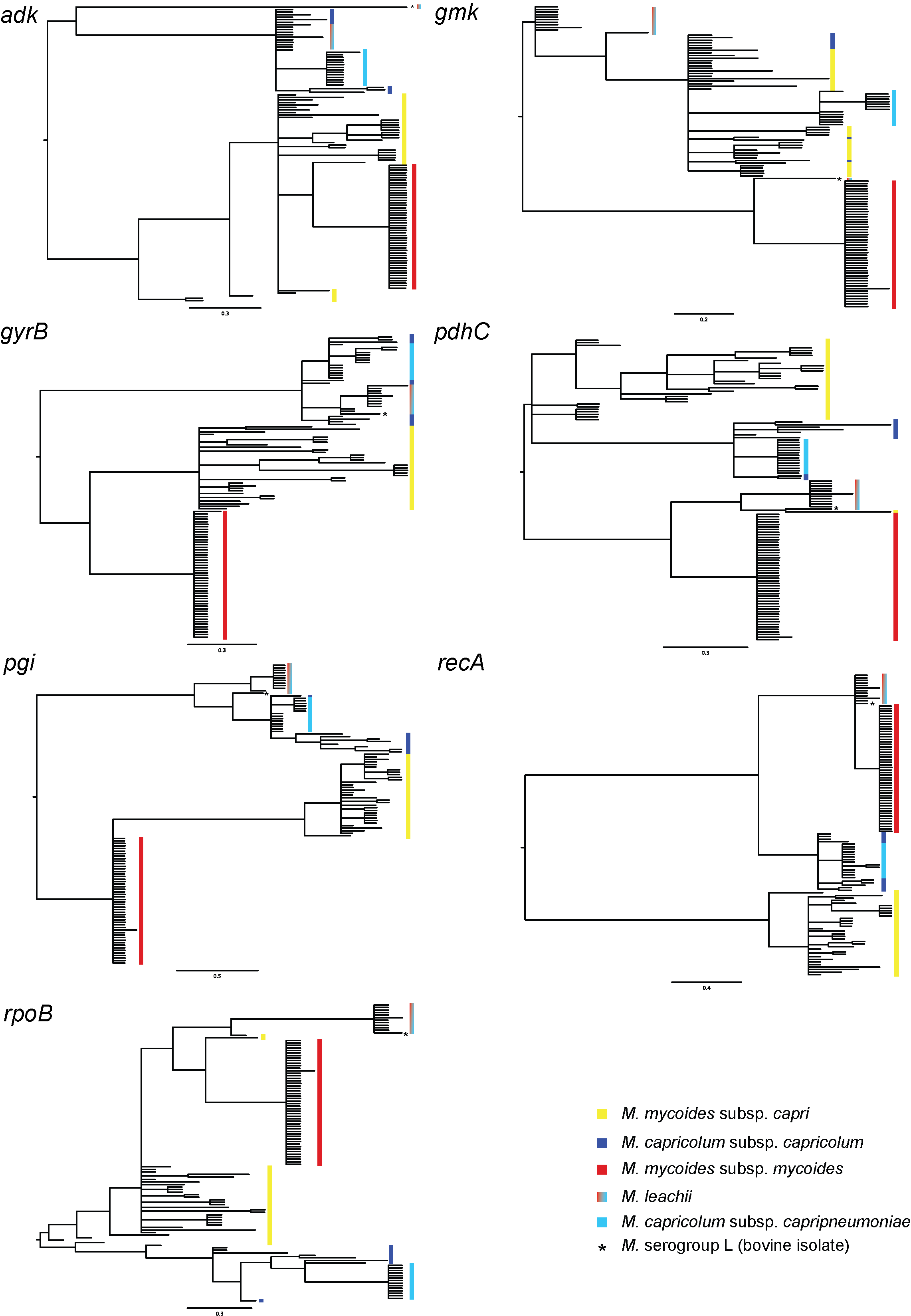

Supplement: Figure S3 — 50% majority consensus tree for each of the seven partial gene sequences as estimated with MrBayes under the GTR+G+I substitution model. (TIF) [file pone.0036150.s003.tif]

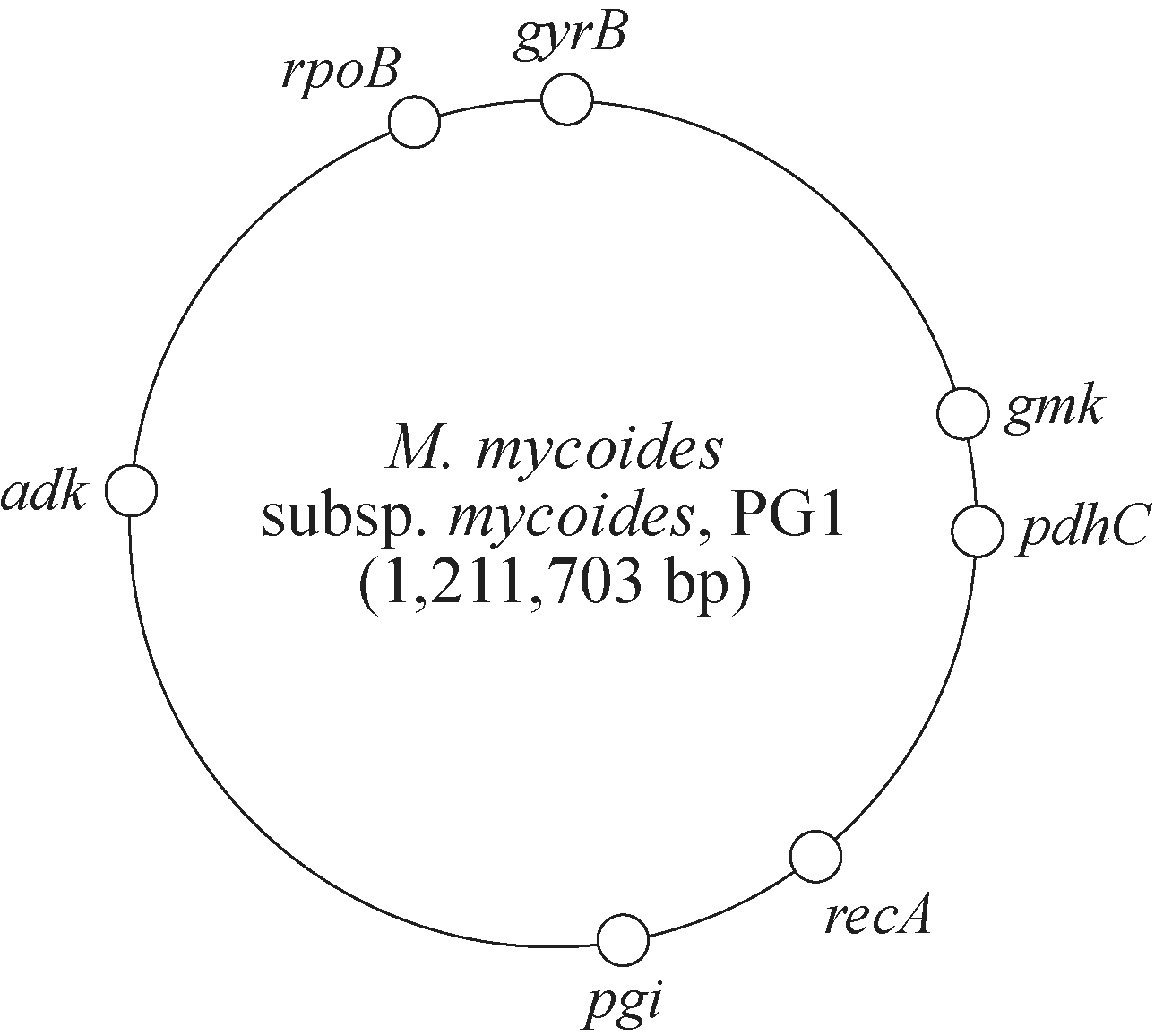

Supplement: Figure S4 — Genomic location of MLST target genes based on the PG1 genome. (TIF) [file pone.0036150.s004.tif]
