# Supplementary material for: The Origin of the ‘Mycoplasma mycoides Cluster’ Coincides with Domestication of Ruminants
Source: PLoS One. 2012 Apr 27;7(4):e36150. doi: 10.1371/journal.pone.0036150 (PMC3338596; doi:10.1371/journal.pone.0036150)
Supplement: Table S1 — Strains used in this study. (DOC) [file pone.0036150.s005.doc]

**Table S1: Strains used in this study.**

| **Strain designation** | **Species** | **Country of origin** | **Year of isolation** | **Host** | **Data Bank / Strain collection** | **Reference** |
| --- | --- | --- | --- | --- | --- | --- |
| ATCC27343 | *Mcc* | USA | 1955 | Goat | GenBank |  |
| 6443.90 | *Mcc* | France | 1990 | Goat | JF |  |
| C47 | *Mcc* | Germany | < 1992 | Sheep | MH |  |
| 4146 | *Mcc* | France | 1980 | Goat | MH |  |
| 609-79 | *Mcc* | France | 1979 | Goat | MH |  |
| B304 | *Mcc* | Portugal | 1984 | Cattle | EB |  |
| 173/87 | *Mcc* | Greece | 1987 | Sheep | JF |  |
| 7714 | *Mcc* | France | 1967 | Goat | CS |  |
| 8086-1 | *Mcc* | France | 1980 | Goat | CS |  |
| 9231-Abomaso | *Mccp* | Ethiopia | 1982 | Goat | EB |  |
| 95043 | *Mccp* | Niger | 1995 | Goat | EB |  |
| 8789 | *Mccp* | Chad | 1987 | Goat | EB |  |
| 91106/550/1 | *Mccp* | Dubai | 1991 | Goat | EB |  |
| M85/98 | *Mccp* | Tanzania | 1998 | Goat | EB |  |
| M79/93 | *Mccp* | Uganda | 1993 | Goat | EB |  |
| 4/2 LC | *Mccp* | Oman | 1988 | Goat | EB |  |
| F38 | *Mccp* | Kenya | 1976 | Goat | JF |  |
| Gabes | *Mccp* | Tunisia | 1980 | Goat | JF |  |
| 87F05 | *Mccp* | Turkey | 2005 | Goat | CS |  |
| D1800/04-1 | *Mccp* | UAE | 2004 | Gazelle | CS |  |
| D1800/04-2 | *Mccp* | UAE | 2004 | Gazelle | CS |  |
| 3535 | *Mccp* | Qatar | 2005 | Mufflon | CS |  |
| GL97P | *Mccp* | Tunisia | 1980 | Goat | CS |  |
| PG50 | *M. leachii* | Australia | 1963 | Cattle | MH |  |
| C2306 | *M. leachii* | Portugal | < 1990 | Cattle | JF |  |
| 4055 | *M. leachii* | France | 1991 | Goat | JF |  |
| PAD3186 | *M. leachii* | India | 1993 | Goat | JF |  |
| FRD424 | *M. leachii* | India | 1993 | Goat | JF |  |
| Calf1 | *M. leachii* | Nigeria | < 1990 | Cattle | JF |  |
| D318b | *M. leachii* | Germany | < 1990 | Cattle | JF |  |
| D424 | *M. leachii* | Germany | < 1990 | Cattle | JF |  |
| CP291 | *M. leachii* | Portugal | 1987 | Goat | JF |  |
| QR1/92 | *M. leachii* | Australia |  |  | JF |  |
| 99/014/6 | *M. leachii* | Australia | 1963 | Calf | JF |  |
| B144P | *M.* serogroup L | USA | 1956 | Cattle | JF |  |
| GM12 | *Mmc* | USA | 1979 | Goat | GenBank |  |
| Y-Goat | *Mmc* | Australia | 1956 | Goat | MH |  |
| LC8065 | *Mmc* | France |  | Goat | JF |  |
| D2482/91 | *Mmc* | Switzerland | 1991 | Goat | JF |  |
| 95010-C1 | *Mmc* | France | 1995 | Goat | JF |  |
| D2083/91 | *Mmc* | Switzerland | 1991 | Goat | JF |  |
| CP271 | *Mmc* | Portugal | 1991 | Goat | JF |  |
| D2503/91 | *Mmc* | Switzerland | 1991 | Goat | JF |  |
| N/108 | *Mmc* | Benin | 1950 | Goat | JF |  |
| WK354/80 | *Mmc* | Switzerland | 1980 | Goat | JF |  |
| G1255.94 | *Mmc* | Berlin | 1994 | Barbary sheep | MH |  |
| G1313.94 | *Mmc* | Germany | 1994 | Barbary sheep | MH |  |
| G1283.94 | *Mmc* | Germany | 1994 | Barbary sheep | MH |  |
| 7302 | *Mmc* | Portugal | < 1994 | Goat | MH |  |
| 7730 | *Mmc* | France | 1994 | Goat | MH |  |
| 136/93 | *Mmc* | Spain | 1993 | Goat | MH |  |
| 80/93 | *Mmc* | Spain | 1994 | Goat | MH |  |
| 153/93 | *Mmc* | Canary Islands | 1993 | Goat | MH |  |
| 83/93 | *Mmc* | Spain | 1993 | Goat | MH |  |
| 171/93 | *Mmc* | Spain | 1993 | Goat | MH |  |
| C260/4 | *Mmc* | Spain | 1993 | Goat | MH |  |
| My-325 | *Mmc* | Croatia | 1986 | Goat | MH |  |
| Kombolcha | *Mmc* | Ethiopia | 1975 | Goat | MH |  |
| 2002-055 | *Mmc* | India |  | Goat | LM |  |
| PG3 | *Mmc* | Turkey | 1950 | Goat | JF |  |
| 1456-11 | *Mmc* | Israel | 1994 | Goat_ | EB |  |
| capri L | *Mmc* | France | 1975 | Goat | MH |  |
| 152/93 | *Mmc* | Grand Canary | 1993 | Goat | MH |  |
| Wi8079 | *Mmc* | Germany | 2009 | Goat | CS |  |
| My-I | *Mmc* | Croatia | 1986 | Goat | CS |  |
| M-5 | *Mmc* | Croatia | 1988 | Goat | CS |  |
| M-18 | *Mmc* | Croatia | 1988 | Goat | CS |  |
| M-29 | *Mmc* | Croatia | 1988 | Goat | CS |  |
| PG1 | *Mmm* | Africa | 1931 | Cattle | JF |  |
| 2059 | *Mmm* | Spain | 1984 | Cattle | JF |  |
| C305 | *Mmm* | Portugal | 1993 | Goat | JF |  |
| B17/90 | *Mmm* | Chad | 1967 | Cattle | JF |  |
| Asmara | *Mmm* | Eritrea | 1970 | Cattle | JF |  |
| 93151 | *Mmm* | Namibia (Kavango) | 1993 | Cattle | JF |  |
| T1/44 | *Mmm* | Tanzania | 1951 | Vaccine strain | JF |  |
| DVZ | *Mmm* | Australia | 1965 | Cattle | JF |  |
| V5 | *Mmm* | Australia | 1936 | Vaccine strain | JF |  |
| B66 | *Mmm* | Kenya | 2000 | Cattle | HW |  |
| B237 | *Mmm* | Kenya | 1997 | Cattle | HW |  |
| PO1967 | *Mmm* | France | 1967 | Cattle | JF |  |
| 6364 | *Mmm* | Spain | 1991 | Cattle | JF |  |
| B773/125 | *Mmm* | Portugal | 1991 | Cattle | JF |  |
| O326 | *Mmm* | Portugal | 1993 | Sheep | JF |  |
| O512 | *Mmm* | Portugal | 1993 | Sheep | JF |  |
| O526 | *Mmm* | Portugal | 1993 | Sheep | JF |  |
| PO 2 | *Mmm* | France | 1980 | Cattle | JF |  |
| 2022 | *Mmm* | France | 1984 | Cattle | JF |  |
| 2091 | *Mmm* | France | 1984 | Cattle | JF |  |
| 2117 | *Mmm* | France | 1984 | Cattle | JF |  |
| 2648 | *Mmm* | Chad | < 2006 | Cattle | JF |  |
| 2055 | *Mmm* | Ethiopia | 1974 | Cattle | JF |  |
| L2 | *Mmm* | Italy | 1993 | Cattle | JF |  |
| 5917 | *Mmm* | Portugal | 1991 | Cattle | JF |  |
| 4426 | *Mmm* | Portugal | 1991 | Cattle | JF |  |
| 4375 | *Mmm* | Senegal | 1977 | Cattle | JF |  |
| 4378 | *Mmm* | Senegal | < 1968 | Cattle | JF |  |
| 5907 | *Mmm* | Portugal | 1995 | Cattle | JF |  |
| KH3J | *Mmm* | Sudan | 1940 | Vaccine strain | JF |  |
| 6471 | *Mmm* | Italy | 1992 | Cattle | JF |  |
| Afadé | *Mmm* | Cameroon | 1968 | Cattle | JF |  |
| 6472 | *Mmm* | Italy | 1992 | Cattle | JF |  |
| Fatick | *Mmm* | Senegal | 1968 | Cattle | JF |  |
| Filfili | *Mmm* | Senegal | 1965 | Cattle | JF |  |
| 87137-10 | *Mmm* | Burkina Faso | 1987 | Cattle | JF |  |
| 91130 | *Mmm* | Central African Republic | 1991 | Cattle | JF |  |
| C11 | *Mmm* | Chad | 1962 | Cattle | JF |  |
| 95014 | *Mmm* | Tanzania | 1995 | Cattle | JF |  |
| T1Sr/50 | *Mmm* | Tanzania | 1951 | Vaccine strain | JF |  |
| Gladysdale | *Mmm* | Australia | 1953 | Cattle | JF |  |
| R575 | *Mmm* | Australia | 1965 | Cattle | JF |  |
| CF1 | *Mmm* | Australia | 1965 | Cattle | JF |  |
| B675/1 | *Mmm* | Portugal | 1993 | Cattle | JF |  |
| B675/2 | *Mmm* | Portugal | 1993 | Cattle | JF |  |
| B675/3 | *Mmm* | Portugal | 1993 | Cattle | JF |  |
| 94111 | *Mmm* | Rwanda | 1994 | Cattle | JF |  |
| 6424 | *Mmm* | Italy | 1992 | Buffalo | JF |  |
| Shawawa | *Mmm* | Botswana | 1995 | Cattle | SG |  |
| B421 | *Mmm* | Portugal | 1993 | Cattle | EB |  |
| G5847 | *M.* sp | Germany | 1993 | Alpine Ibex | MH |  |
| G5813/1+2 | *M.* sp | Germany | 1993 | Alpine Ibex | MH | MH |
| G1650 | *M.* sp | Germany | 1994 | Alpine Ibex | MH |  |
| G1705 | *M.* sp | Germany | 1994 | Alpine Ibex | MH | MH |
| 8756-C13 | *M.* sp | USA | < 1987 | Rocky Mountain Goat | MH |  |

*Mmc* - *Mycoplasma mycoides* subsp. *capri*, *Mcc* - *Mycoplasma capricolum* subsp. *capricolum*, *Mccp – Mycoplasma capricolum* subsp. *capripneumoniae*, *Mmm* - *Mycoplasma mycoides* subsp. *mycoides*, *M.* sp. - unassigned *Mycoplasma* species

CS – Christiane Schnee, EB - Erik Bonkcam-Rudloff, JF – Joachim Frey, LM – Lucia Manso-Silvan, MH – Martin Heller, HW - Hezron Wesonga

**References**

1. Cordy DR, Adler HE, Yamamoto R (1955) A pathogenic pleuropneumonia-like organism from goats. Cornell Vet 45: 50-68.

2. Thiaucourt F, Lorenzon S, David A, Breard A (2000) Phylogeny of the *Mycoplasma mycoides* cluster as shown by sequencing of a putative membrane protein gene. Vet Microbiol 72: 251-268.

3. Schnee C, Heller M, Jores J, Tomaso H, Neubauer H (2011) Assessment of a novel multiplex real-time PCR assay for the detection of the CBPP agent *Mycoplasma mycoides* subsp. *mycoides* SC through experimental infection in cattle. BMC veterinary research 7: 47.

4. Christiansen G, Ernø H (1990) RFLP in rRNA genes of *Mycoplasma capricolum*, the caprine F38-like group and the bovine serogroup 7. Zentralblatt für Bakteriologie 20: 479-488.

5. Perreau P, Breard A (1979) La mycoplasmose caprine a *M. capricolum*. Comp Immunol Microbiol Infect Dis 2: 87-97.

6. Manso-Silvan L, Perrier X, Thiaucourt F (2007) Phylogeny of the *Mycoplasma mycoides* cluster based on analysis of five conserved protein-coding sequences and possible implications for the taxonomy of the group. Int J Syst Evol Microbiol 57: 2247-2258.

7. Thiaucourt F, Breard A, Lefevre PC, Mebratu GY (1992) Contagious caprine pleuropneumonia in Ethiopia. The Veterinary record 131: 585.

8. Pettersson B, Bolske G, Thiaucourt F, Uhlen M, Johansson KE (1998) Molecular evolution of *Mycoplasma capricolum* subsp. *capripneumoniae* strains, based on polymorphisms in the 16S rRNA genes. Journal of bacteriology 180: 2350-2358.

9. Kusiluka LJ, Ojeniyi B, Friis NF, Kokotovic B, Ahrens P (2001) Molecular analysis of field strains of *Mycoplasma capricolum* subspecies *capripneumoniae* and *Mycoplasma mycoides* subspecies *mycoides*, small colony type isolated from goats in Tanzania. Vet Microbiol 82: 27-37.

10. Djordjevic SR, Forbes WA, Forbes-Faulkner J, Kuhnert P, Hum S, et al. (2001) Genetic diversity among *Mycoplasma* species bovine group 7: clonal isolates from an outbreak of polyarthritis, mastitis, and abortion in dairy cattle. Electrophoresis 22: 3551-3561.

11. Vilei EM, Korczak BM, Frey J (2006) *Mycoplasma mycoides* subsp. *capri* and *Mycoplasma mycoides* subsp. *mycoides* LC can be grouped into a single subspecies. Vet Res 37: 779-790.

12. Atalaia V, Machado M, Frazao FF (1987) Patologia dos pequenos ruminantes infecco ̃es em ovinos e caprinos, originadas pelo micoplasma do grupo 7, Leach (Pg. 50). Rep Trab LNIV 19: 55-60.

13. Stipkovits L, El-Ebeedy A (1977) Biochemical and serological studies of avian mycoplasmas. Zentralblatt fur Veterinarmedizin Reihe B Journal of veterinary medicine Series B 24: 218-230.

14. DaMassa AJ, Brooks DL, Adler HE (1983) Caprine mycoplasmosis: widespread infection in goats with *Mycoplasma mycoides* subsp *mycoides* (large-colony type). American journal of veterinary research 44: 322-325.

15. Laws L (1956) A pleuropneumonia-like organism causing peritonitis in goats. Aust Vet J 32: 326-329.

16. Thiaucourt F, Manso-Silvan L, Salah W, Barbe V, Vacherie B, et al. (2011) *Mycoplasma mycoides*, from "*mycoides* Small Colony" to "*capri*". A microevolutionary perspective. BMC genomics 12: 114.

17. Griffin RM (1969) Antigenic relationships among strains of *Mycoplasma mycoides* var. *mycoides*, *M. capri* and *M. laidlawii* revealed by complement-fixation tests. J Gen Microbiol 57: 131-142.

18. Dedieu L, Mady V, Lefevre PC (1994) Development of a selective polymerase chain reaction assay for the detection of *Mycoplasma mycoides* subsp. *mycoides* S.C. (contagious bovine pleuropneumonia agent). Vet Microbiol 42: 327-339.

19. Woubit S, Lorenzon S, Peyraud A, Manso-Silvan L, Thiaucourt F (2004) A specific PCR for the identification of *Mycoplasma capricolum* subsp. *capripneumoniae*, the causative agent of contagious caprine pleuropneumonia (CCPP). Veterinary microbiology 104: 125-132.

20. de la Fe C, Assuncao P, Rosales RS, Antunes T, Poveda JB (2006) Characterisation of protein and antigen variability among *Mycoplasma mycoides* subsp. *mycoides* (LC) and *Mycoplasma agalactiae* field strains by SDS-PAGE and immunoblotting. Vet J 171: 532-538.

21. Tully JG, Barile MF, Edward DG, Theodore TS, Erno H (1974) Characterization of some caprine mycoplasmas, with proposals for new species, *Mycoplasma capricolum* and *Mycoplasma putrefaciens*. Journal of general microbiology 85: 102-120.

22. Rapaport E, Flitman-Tene R, Yogev D, Levisohn S (1999) Outbreaks of contagious agalactia in Israel: Clinical and epizootiological aspects. In: Stipkovits L, Rosengarten R, Frey J, editors. Mycoplasmas of Ruminants: pathogenicity, diagnostics, epidemiology and molecular genetics. Luxembourg: European Commission, European Communities Official Publications Office. pp. 120-123.

23. Cheng X, Nicolet J, Poumarat F, Regalla J, Thiaucourt F, et al. (1995) Insertion element IS1296 in *Mycoplasma mycoides* subsp. *mycoides* small colony identifies a European clonal line distinct from African and Australian strains. Microbiology 141 ( Pt 12): 3221-3228.

24. Yaya A, Manso-Silvan L, Blanchard A, Thiaucourt F (2008) Genotyping of *Mycoplasma mycoides* subsp. *mycoides* SC by multilocus sequence analysis allows molecular epidemiology of contagious bovine pleuropneumonia. Vet Res 39: 14.

25. Jores J, Nkando I, Sterner-Kock A, Haider W, Poole J, et al. (2008) Assessment of *in vitro* interferon-gamma responses from peripheral blood mononuclear cells of cattle infected with *Mycoplasma mycoides* ssp. *mycoides* small colony type. Vet Immunol Immunopathol 124: 192-197.

26. Bischof DF, Vilei EM, Frey J (2006) Genomic differences between type strain PG1 and field strains of *Mycoplasma mycoides* subsp. *mycoides* small-colony type. Genomics 88: 633-641.

27. Bothelo A (2011) Personel Communication.

28. Varela F, Inacio J, Botelho A (2010) Molecular diversity assessed by VNTR and IS1296 typing of historical *Mycoplasma mycoides* subsp. *mycoides* SC strains. Vet Microbiol.

29. Vilei EM, Frey J (2004) Differential clustering of *Mycoplasma mycoides* subsp. *mycoides* SC strains by PCR-REA of the bgl locus. Vet Microbiol 100: 283-288.

30. Goncalves R, Regalla J, Nicolet J, Frey J, Nicholas R, et al. (1998) Antigen heterogeneity among *Mycoplasma mycoides* subsp. *mycoides* SC isolates: discrimination of major surface proteins. Vet Microbiol 63: 13-28.

31. Gorton TS, Barnett MM, Gull T, French RA, Lu Z, et al. (2005) Development of real-time diagnostic assays specific for *Mycoplasma mycoides* subspecies *mycoides* Small Colony. Vet Microbiol 111: 51-58.
