# Supplementary material for: The Origin of the ‘Mycoplasma mycoides Cluster’ Coincides with Domestication of Ruminants
Source: PLoS One. 2012 Apr 27;7(4):e36150. doi: 10.1371/journal.pone.0036150 (PMC3338596; doi:10.1371/journal.pone.0036150)
Supplement: Table S2 — Target genes, primer sequences and trimming region used for MLST of the ‘M. mycoides cluster’. (DOC) [file pone.0036150.s006.doc]

**Table S2: Target genes, primer sequences and trimming region used for MLST of the *‘M. mycoides cluster’*.**

| Gene | Forward Primer (5’-3’) | Reverse Primer (5’-3’) | Amplicon size in bp |
| --- | --- | --- | --- |
| *adk* | AGCACCTGGTTGTGGAAAAG | TTTGATTAAAAACTTGTTCAGCTG | 599 |
| *gmk* | TTTGCAAAAGCCATTGTTAATG | AAATCACCATGTCCTAGAACTTTG | 605 |
| *gyrB* | GAAGACATTGAAGTTGAAATGGC | ACCATCAACATCTGCATCAGTC | 765 |
| *pdhC* | CCAATGAATGGTGTTAGAAAAGC | AATTAAAAATCTTCCAGCATCTGC | 633 |
| *pgi* | TGGAAGGTTTTCAGTTTTAACTCC | TCAACTCCAGGTTGATTAAATGG | 654 |
| *recA* | AATGTCGAAACTTTTTCATCAGG | GGTTGCAGTTTTAAAAGGAATAGC | 672 |
| *rpoB* | ATTAACAGCTTTAGGATCTGGTGG | CAATTCTTTCACTCATAACGATCG | 860 |
| Target genes and corresponding sequencing primers | | | |
| *Gene* | Forward Primer (5’-3’) | Reverse Primer (5’-3’) |  |
| *adk* | TGGAAAAGGAACACAAGCAG | TGATAAATCATCTGCTTTAATTTCA |  |
| *gmk* | GCAAAAGCCATTGTTAATGA | CCATGTCCTAGAACTTTGTGAT |  |
| *gyrB* | CATTGAAGTTGAAATGGCACT | TCAACATCTGCATCAGTCATAAT |  |
| *pdhC* | AAAAGCAATGACAAAATCACA | CTTCCAGCATCTGCACCAT |  |
| *pgi* | AACTCCAGTTGGGATTTTTCC | TCCAGGTTGATTAAATGGATTA |  |
| *recA* | CGAAACTTTTTCATCAGGTTCA | CAGTTTTAAAAGGAATAGCAGTTT |  |
| *rpoB* | AGCTTTAGGATCTGGTGGTTT | CATAACGATCGCATCTTCAAA |  |
| Target genes and trimming sequences used | | | |
| *Gene* | Start Sequence | Stop Sequence | Size in bp |
| *adk* | AATAAATTARATTTTATT | AAATTTATTRAAATTAAA | 519 |
| *gmk* | GGWACTCCTAGAAAATAT | ACTTATTTAATTAAATTA | 498 |
| *gyrB* | TTTGTAAATAAYATTAAT | GARGCTAGAGCYTTTTCR | 585 |
| *pdhC* | AAAAAYACTGAYATTACT | AAAAGATTTATAATGCCA | 495 |
| *pgi* | GGKGCTTTAAAAGCMAAA | CARTTTGGWTAYTTRGTT | 501 |
| *recA* | AARGGAAGAATTATTGAA | TCAGAAATTATTGGWAAT | 549 |
| *rpoB* | ATTTGTCCTATTGAAACW | ATTATTGCTGATGGWCCW | 669 |

*adk* - adenylate kinase, *gmk* - guanylate kinase, *gyrB* - DNA gyrase subunit B, *pdhC* - dihydrolipoamide S-acetyltransferase, *pgi* - glucose-6-phosphate isomerase, *recA* - recombination protein, *rpoB* - RNA polymerase b-subunit
