# Supplementary material for: The Origin of the ‘Mycoplasma mycoides Cluster’ Coincides with Domestication of Ruminants
Source: PLoS One. 2012 Apr 27;7(4):e36150. doi: 10.1371/journal.pone.0036150 (PMC3338596; doi:10.1371/journal.pone.0036150)
Supplement: Table S3 — Bayesian MCMC estimates of time to the most recent common ancestor in years before present. (DOC) [file pone.0036150.s007.doc]

Table S3: Bayesian MCMC estimates of time to the most recent common ancestor in years before present.

|  | Root | *M. capricolum* | *Mmc* | *Mmm* | *M. leachii* |
| --- | --- | --- | --- | --- | --- |
| Mean | 10097 | 2331 | 3450 | 218 | 2998 |
| Standart error of mean | 240 | 56 | 82 | 4 | 70 |
| Median | 9305 | 2148 | 3171 | 191 | 2761 |
| 95% HPD lower | 4089 | 937 | 1398 | 91 | 1175 |
| 95% HPD upper | 17974 | 4201 | 6201 | 414 | 5421 |

HPD – high posterior density, *M.* – *Mycoplasma*, Mmc – *Mycoplasma mycoides* subsp. *capri*, Mmm - *Mycoplasma mycoides* subsp. *capri*
